# Design of Multi-Functional Superhydrophobic Coating *via* Bacterium-Induced Hierarchically Structured Minerals on Steel Surface

**DOI:** 10.3389/fmicb.2022.934966

**Published:** 2022-06-16

**Authors:** Yiwen Zhang, Tao Liu, Jian Kang, Na Guo, Zhangwei Guo, Jinghao Chen, Yansheng Yin

**Affiliations:** ^1^College of Ocean Science and Engineering, Shanghai Maritime University, Shanghai, China; ^2^School of Mechanical Engineering, Beijing Institute of Petrochemical Technology, Beijing, China; ^3^State Key Laboratory of RAL, Northeastern University, Shenyang, China; ^4^Engineering Technology Research Center for Corrosion Control and Protection of Materials in Extreme Marine Environment, Guangzhou Maritime University, Guangzhou, China

**Keywords:** biomineralization, superhydrophobic, self-cleaning, anti-corrosion, mechanically robust

## Abstract

The fabrication of an eco-friendly, multi-functional, and mechanically robust superhydrophobic coating using a simple method has many practical applications. Here, inspired by shell nacre, the micro- or nano-scale surface roughness that is necessary for superhydrophobic coatings was formed *via Bacillus subtilis*–induced mineralization. The biomineralized film coated with hexadecyltrimethoxysilane (HDTMS) exhibited superhydrophobicity with water contact angles of 156°. The biomimetic HDTMS/calcite-coating showed excellent self-cleaning, anti-icing, and anti-corrosion performances. Furthermore, mechanically robust superhydrophobicity could be realized by hierarchically structured biomineralized surfaces at two different length scales, with a nano-structure roughness to provide water repellency and a micro-structure roughness to provide durability. Our design strategy may guide the development of “green” superhydrophobic coatings that need to retain effective multi-functional abilities in harsh marine environments.

## Introduction

Superhydrophobic coatings with high contact angles (CA > 150°) and low-sliding angles (SA > 10°) of water have attracted much attention because of their many potential applications (Nakajima et al., 1999), such as self-cleaning (Guldin et al., 2013; Stieberova et al., 2017), oil/water separation (Ye et al., 2017; Tang et al., 2021), anti-fogging (Chevallier et al., 2011; Chang et al., 2012), anti-corrosion (Liu et al., 2007, 2010), anti-biofouling (Lopez et al., 2016; Baidya et al., 2017), and anti-icing (Emelyanenko et al., 2017; Wang et al., 2017). According to the Wenzel and Cassie–Baxter models, materials with micro- or nano-scale surface roughness and low-surface energies are both critical to the preparation of superhydrophobic surfaces (Wenzel, 1936; Wang and Jiang, 2007). To date, low-surface-energy materials are mostly organic compounds containing fluorine and/or silicon (Sheen et al., 2008; Manca et al., 2009; Yang et al., 2010). However, designing a suitable surface roughness is one of the most challenging scientific and technological bottlenecks in preparing superhydrophobic surfaces (Wang et al., 2006). Many fabrication methods are expensive, complicated, and environmentally hazardous and yield materials with poor-mechanical stability (Phuong et al., 2019). Therefore, a simple and eco-friendly method to prepare a rough surface with corrosion and wear resistance properties is important.

Inspired by shell nacre, biomineralization may be a perfect method for self-assembling–layered inorganic crystals with precise control over the arrangement, shape, architecture, and size (Qi et al., 2002). It has been reported that over 200 bacteria strains, including *B. subtilis*, *Pseudomonas* spp., and *Azotobacter* spp., are capable of inducing calcium carbonate precipitation in soil and seawater (Liu et al., 2018). Biomineralization is a complex process that relies on the environmental physiochemical conditions and the metabolism of the microorganism. Calcium carbonate precipitation requires the nucleation site, the presence of Ca^2+^, and proper pH. Extracellular polymeric substance (EPS) produced by microorganisms can be complex with metal ions such as Ca^2+^ and Mg^2+^ in the environment, forming organic–inorganic composite biomineralized films (Yu et al., 2021). As a microorganism with the capacity for ammonification, *B. subtilis* can decompose proteins and various nitrogen-containing organic substances to ammonium ions (NH^+^) and bicarbonate (HCO^–^), effectively, increasing the pH of the medium (Hoffmann et al., 1998). Biomineralized films have some advantages that other rough surfaces normally lack, such as anti-corrosion, anti-wear, and strong adhesion (Guo et al., 2021). Our previous work showed that the biomineralized crystal induced by *Pseudoalteromonas lipolytica* and *B. subtilis* exhibited trapezoidal pillars with a hierarchical structure with micro- and nano-scale features (Liu et al., 2018). Moreover, the barrier protection imparted by this biomineralized film was comparable to or even better than some previously reported organic anti-corrosion coatings (Guo et al., 2022). In addition, the adhesion of biomineralization films is so strong that they cannot easily be scraped off of steel surfaces with a knife (Guo et al., 2019a,b). Without any manual intervention, natural biomineralized films may intelligently provide micro-/nano-rough surfaces that are necessary to prepare superhydrophobic coatings. Recently, some researchers have prepared biomimetic mineralized coatings *via* various chemical methods. However, these methods usually involve a complicated multi-step process, as well as high-temperature treatment (Hu and Deng, 2010; Wang et al., 2011; Atta et al., 2016). Moreover, biomimetic mineralized coatings endowing good corrosion and wear resistance properties have seldom been reported. For some coatings, a slight finger touch can fatally destroy the micro-/nano-structures of the superhydrophobic surfaces, not to mention heavy-duty friction. The damage of the micro-/nano-structures enhances the solid–liquid contact area, leading to the adhesion of water droplets on the coatings and finally, a loss of the superhydrophobicity (Peng and Garnier, 2010).

In this study, hierarchical micro-/nano-structured biomineralized films were first formed on the surfaces of steel *via B. subtilis*–induced mineralization followed by the spraying of hexadecyltrimethoxysilane (HDTMS). After this, a non-fluorinated, mechanically robust superhydrophobic coating for anti-icing, self-cleaning, and anti-corrosion in marine environments was obtained. Furthermore, the superhydrophobic coating showed resistance to an acetic acid salt spray test, overcoming one of the biggest shortcomings of carbonate mineralized films. Therefore, this study is expected to inspire new ideas for preparing superhydrophobic coatings for harsh environment applications.

## Experimental

### Materials

Hexadecyltrimethoxysilane, acridine orange, methylene blue, anhydrous ethanol, acetone, CuCl_2_⋅2H_2_O, NaCl, and acetic acid were provided by Sigma-Aldrich. The steel used in this study was obtained from Baosteel Inc., China. The alloy element composition of the steel is (wt.%): 1.5 Mn, 0.70 Ni, 0.20 Si, 0.15 Ti, 0.04 Al, 0.02 Nb, 0.055 C, and Fe balance. The steel was cut into squares to create 10 mm × 10 mm working surfaces, and the steel coupons were polished with 400- to 1,200-mesh silicon carbide paper in sequence. The coupons were then cleaned by ultrasonication in alcohol and acetone, washed with H_2_O, and dried in N_2_. Before immersion in the medium, all the coupons were sterilized under ultraviolet (UV) light for 30 min.

### Preparation of Hexadecyltrimethoxysilane/Calcite Coating

**FIGURE 1:**
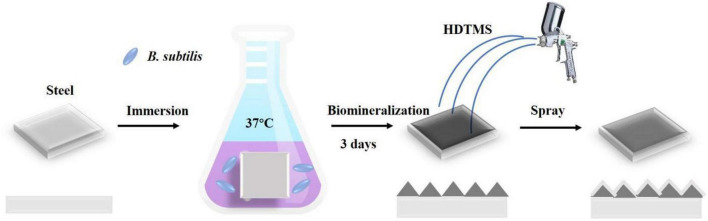
Schematic of fabrication process for the superhydrophobic hexadecyltrimethoxysilane (HDTMS)/calcite coating.

*Bacillus subtilis* was cultured in 2216E medium (5.0 g/L peptone, 1.0 g/L yeast extract, 0.01 g/L ferric citrate, 19.45 g/L NaCl, 5.98 g/L MgCl_2_, 3.24 g/L Na_2_SO_4_, 1.8 g/L CaCl_2_, 0.55 g/L KCl, 0.16 g/L Na_2_CO_3_, 0.08 g/L KBr, 0.034 g/L SrCl_2_, 0.022 g/L H_3_BO_3_, 0.004 g/L NaSiO_3_, 0.0024 g/L NaF, 0.0016 g/L NH_4_NO_3_, and 0.008 g/L Na_2_HPO_4_ dissolved in deionized water) on a rotary shaker at 150 rpm and 36°C. Approximately 0.25 ml of overnight culture was added to the container (final bacterial density ∼1 × 10^7^ CFU/ml), which were incubated in a shaking incubator at 150 rpm for up to 3 days at 36°C. At least three independent experiments were conducted and evaluated. The coupons (five replicated) were immersed in the culture solution to prepare biomineralized films. After 3 days of incubation, a calcite biomineralized film on the steel coupon surface was obtained. After rinsing with deionized water, the biomineralized film was then dried at room temperature or rapidly dried at 50°C. Then, HDTMS was sprayed three times for 30 s each on the surface of the biomineralized film to obtain the superhydrophobic HDTMS/calcite coating. The distance between the hand-held spray gun and the coupon surface is approximately 30 cm, as shown in Figure 1.

### Characterization

The surface morphologies and elemental distributions were observed using scanning electron microscopy with energy-dispersive X-ray spectroscopy (SEM-EDS, Hitachi TM4000Plus, Japan). The cross-sectional topography and corresponding energy spectrum distribution were investigated by cyro-focused ion beam scanning electron microscopy (FIB–SEM) (Crossbeam 350, Zeiss, Germany). To determine the crystal structure of the biomineralized film, atomic force microscopy (AFM; Dimension Icon ScanAsyst, Bruker) analysis was carried out on the calcite crystals. The phase identification of the biomineralized film was achieved by X-ray diffraction (XRD) using a PANalytical X’Pert PRO XRD system (Netherlands) at 40 kV and 10 mA, with a Cu Kα radiation source and scanning at a rate of 0.26° s^–1^ within the 2θ range of 20–90°. Fourier-transform infrared spectroscopy (FTIR, Bruker Vertex 70, Germany) was also used to identify the HDTMS, which was successfully sprayed on the biomineralized film. Furthermore, the sample cross-sectional morphologies and elemental distributions were investigated using focused ion beam scanning electron microscopy (FIB–SEM, Zeiss Crossbeam 550, Germany). The biofilm on the copper surface was fluorescent-stained using a LIVE/DEAD Biofilm Viability Kit (Invitrogen, Thermo Fisher Scientific, United States) and then observed *via* laser scanning confocal microscopy (LSCM, Leica-TCS SP8 STED 3X, Germany).

### Self-Cleaning Behavior

A self-cleaning test was used to investigate the self-cleaning performances of the coatings. Two typical pollutants, methylene blue and soil, were used to pollute the steel, biomineralized film, and HDTMS/calcite coating. First, soil particles were scattered on the steel, biomineralized film, and HDTMS/calcite coating surfaces. Subsequently, the surfaces polluted with soil were washed with a methylene blue solution (0.25 wt %) dropwise, and the visual inspection of self-cleaning performance was evaluated by determining the amount of remaining residual pollutants on the surfaces.

### Anti-icing Behavior

The anti-icing behavior of the steel, biomineralized film, and HDTMS/calcite coating was investigated at −20°C. The samples were placed in the refrigerator for 30 min and pre-cooled to −20°C. Then, 50 μl of methylene blue solution was dropped on the surface of each sample. During freezing, the morphologies of the methylene blue solution droplets were also recorded with the icing time.

### Mechanical Stability

To test the mechanical stability of the HDTMS/calcite coating, the coating was placed in contact with 2,000-mesh sandpaper and was rubbed back and forth for 200 cycles at a distance of 40 cm with 9.8 kPa of pressure. Afterward, water droplet CAs analysis was conducted. The CAs were conducted using a JC2000A CA system at ambient temperature.

### Anti-corrosion Behavior

A platinum plate, saturated calomel electrode, and working electrode were used in a traditional three-electrode system for electrochemical testing, and a 500-ml volumetric flask was used as the electrochemical cell. Electrochemical impedance spectroscopy (EIS) test was performed by an electrochemical workstation (Auto Lab, PGSTAT302, Switzerland) with a test frequency of 10^–2^–10^5^ Hz and a sinusoidal voltage of ± 10 mV using the ZSimpWin analysis software. Between the two EIS tests, an acetic acid salt spray test was conducted to evaluate the anti-corrosion behavior of the HDTMS/calcite coating. In addition, CuCl_2_⋅2H_2_O (0.26 g/L) was added to the 5.0% NaCl solution, and the pH of the solution was adjusted to 3.0 using acetic acid. The samples were subjected to morphology analysis after total testing of 120 h.

## Results and Discussion

**FIGURE 2:**
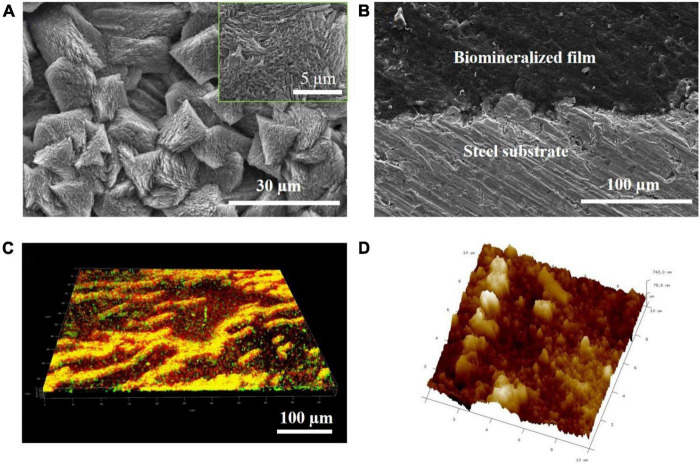
*Bacillus subtilis* strain–induced calcite biomineralization on the steel surface. **(A)** Scanning electron microscopy (SEM) image of the morphology of the biomineralized film induced by the *B. subtilis* strain on day 3. **(B)** Cross-sectional SEM images of the biomineralized film induced by the *B. subtilis* strain on day 3. **(C)** LSCM images of the biofilm cells stained with the LIVE/DEAD Biofilm Viability kit on the steel surface on day 3 of the immersion test. Live cells are shown as green, dead cells as red, and partially damaged/dead cells as yellow. **(D)** Atomic force microscopy (AFM) image of the biomineralized film induced by the *B. subtilis* strain on day 3. The scanning size was 10 μm × 10 μm.

To elucidate the hierarchical structure of the biomineralized film, cross-sectional SEM, AFM, and LSCM were performed at multiple locations on the steel sample on day 3. Figure 2A shows that a relatively dense mineral-like film-featuring trigonal structures were formed on the steel surface. The magnified inset in Figure 2A shows that each trigonal structure was formed by layers of small needle-like pillars growing in different directions. Furthermore, the interface bonding between the biomineralized film and the steel substrate was close, indicating that the adhesion of the biomineralized film may be very sturdy (Figure 2B). In addition, almost no defects were evident in the cross-section of the film, exhibiting the perfect structure of natural biomineralization. Figure 2C reveals that a large number of *B. subtilis* cells adhered to the surface of the biomineralized film, and many dead cells accumulated together. The accumulation of bacteria does not have a significant effect on the surface morphology because these bacteria are very soft compared with the mineralized products. After culturing in the marine medium with *B. subtilis* for 3 days, a surface with micro- or nano-scale roughness was formed on the steel substrate, as shown in the AFM image (Figure 2D). The smaller humps were several nanometers in size, while the larger ones were several hundred nanometers in size. These results indicated that bacterium-induced hierarchically structured minerals formed on the steel surface after three days of immersion, which satisfied one of the necessary conditions for forming a superhydrophobic surface.

**FIGURE 3:**
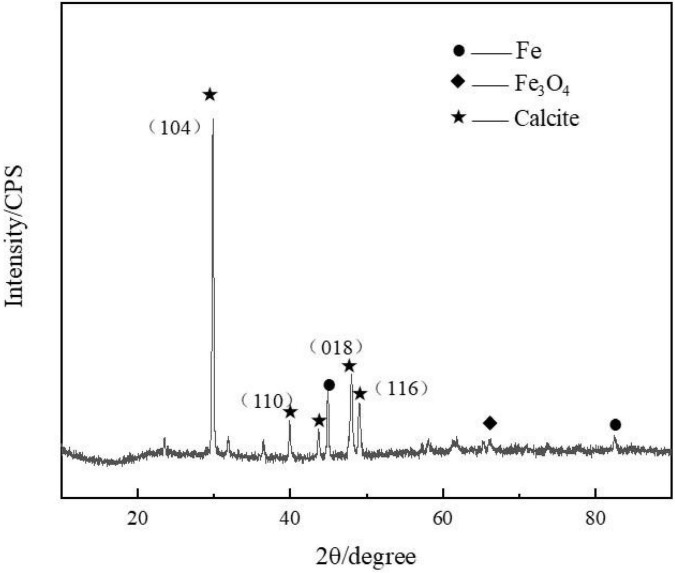
Presence of calcite (mainly calcium carbonate) was confirmed by the X-ray diffraction (XRD) spectrum measured on the steel specimens after 3 days of immersion in marine broth with *Bacillus subtilis* strain.

The biomineralization process enabled the formation of the calcite-type CaCO_3_ minerals on the steel surface, which produced three major diffraction peaks of 29, 49, and 48° (Figure 3). This has been reported previously for calcium carbonate (Liu et al., 2018).

**FIGURE 4:**
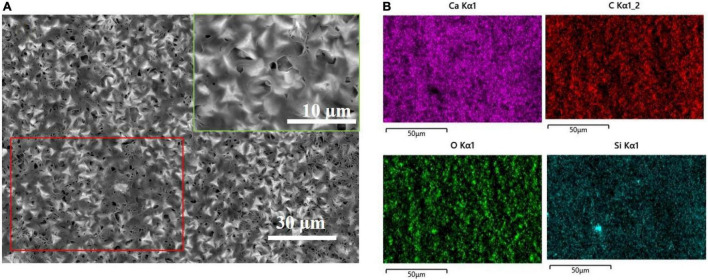
After spraying the biomineralized film with HDTMS. **(A)** Low and high (green frame) magnification SEM images of HDTMS/calcite coating and **(B)** the correspondent (red frame) energy-dispersive X-ray spectroscopy (EDS) mapping (Ca, C, O, and Si).

After spraying HDTMS on the biomineralized film, the calcite morphology with trigonal structures was clearly visible in the SEM image, indicating that the HDTMS film sprayed on the calcite/steel surface was very thin. Figures 4A,B shows the distributions of Ca and Si on the coating surface, revealing that a homogeneous HDTMS coating was formed on the biomineralized film.

**FIGURE 5:**
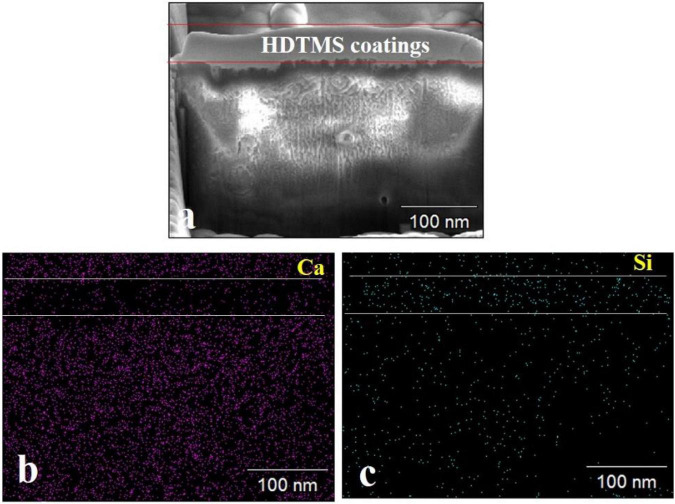
Cross-sectional SEM images of the HDTMS/calcite coating and the distribution of relevant elements. **(a)** Cross-sectional SEM images of the HDTMS/calcite coating. **(b)** Distributions of Ca and **(c)** distributions of Si of the HDTMS/calcite coating.

FIB–SEM analysis was used to observe the cross-sections of the HDTMS/calcite coating. Figure 5a shows that the thickness of the HDTMS coating was approximately 70–80 nm. From the elemental distributions (Ca and Si), we determined that the HDTMS penetrated and filled the defects located in the biomineralized layer, thereby, forming a tight interface (Figures 5B,C).

**FIGURE 6:**
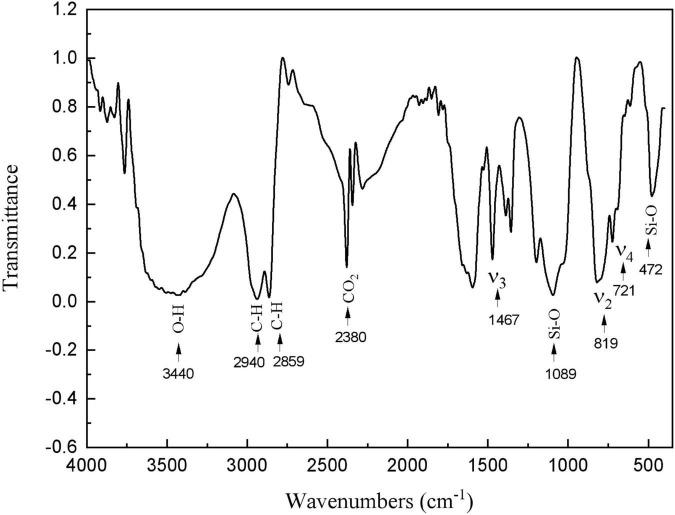
Fourier transform infrared spectroscopy (FTIR) spectra of HDTMS/calcite coating.

The presence of calcite and HDTMS was further confirmed by FTIR (Figure 6). A unique fingerprint of the spectrum can be identified as calcite, containing ν_2_, ν_3_, and ν_4_ peaks at 1,467, 819, and 721 cm^–1^ (Liu et al., 2018). Furthermore, vibration peaks of organic matter were also observed in the infrared spectra. Figure 6 shows the symmetric stretching and bending vibration bands for Si-O at 1,089 and 472 cm^–1^, respectively (Zhou et al., 2018; Almajed et al., 2021). The bands corresponded to the stretching vibrations of the C-H groups, which appeared at 2,940 and 2,859 cm^–1^ (Almajed et al., 2021; Banerjee and Chakraborty, 2021). The 3,440 cm^–1^ peak was due to O–H bonds (Liu et al., 2018). The 2,380 cm^–1^ peak was due to the CO_2_ in the testing environment (Almajed et al., 2021). These results suggest that this coating was an organic–inorganic hybrid film that was composed of organosiloxane and calcite.

**FIGURE 7:**
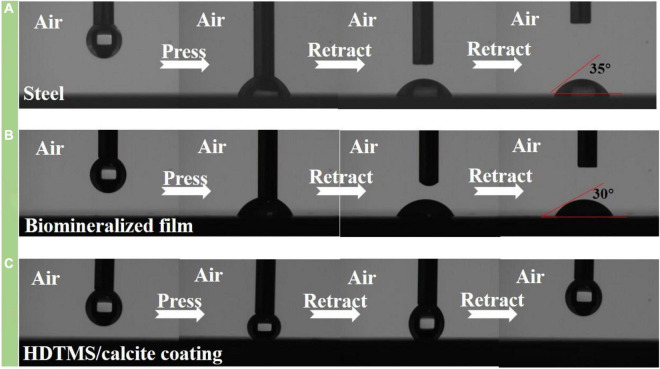
Images of adhesion behavior of water droplets on **(A)** steel, **(B)** biomineralized film, and **(C)** HDTMS/calcite coating.

After micro- or nano-scale-surface-roughness and low-surface-energy materials were realized, the hydrophobic properties of HDTMS/calcite coating were investigated by measuring the CAs of water. As the control groups, the CAs of the steel surface and biomineralized film were also measured. Figures 7A,B show that the CAs of water on the steel and biomineralized film surfaces were 35 and 30°, respectively, indicating that the biomineralized film was hydrophilic. However, due to the superhydrophobicity of the HDTMS/calcite coating surface (CA > 151°), a water droplet barely adhered to the surface and followed the needle tip away from the surface when the tip was lifted (Figure 7C).

**FIGURE 8:**
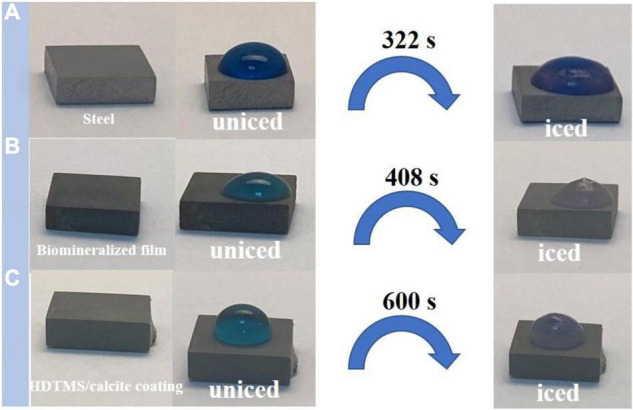
Photographs of water droplets on **(A)** steel, **(B)** biomineralized film, and **(C)** HDTMS/calcite coating under low-temperature conditions (–20°C).

The anti-icing performance of the HDTMS/calcite coating was investigated by a static icing experiment in a refrigerator (−20°C). During the static icing test, 40 μl of methylene-blue-stained water droplets were placed on the steel, biomineralized film, and HDTMS/calcite coating surfaces, and the freezing process and time were recorded with a camera. On the bare steel, the water droplets were completely frozen after 322 s (Figure 8A), and on the biomineralized film, the water droplets froze completely after 408 s (Figure 8B). For the HDTMS/calcite coating (Figure 8C), the droplets froze after 600 s, which was attributed to two factors. First, the contact area between the water droplets and the HDTMS/calcite coating was small, which decreased ice nucleation and limited the expansion of the ice film. Second, heat conduction was prevented due to the air cushion between the water droplets and the HDTMS/calcite coating.

**FIGURE 9:**
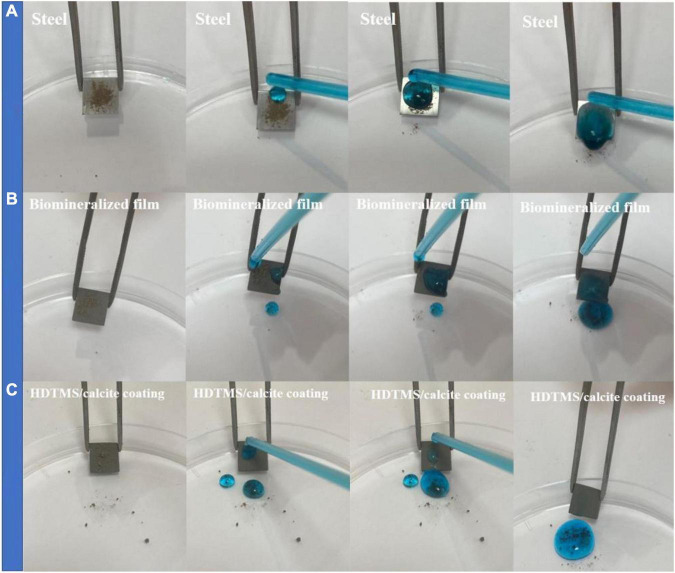
Self-cleaning properties on **(A)** steel, **(B)** biomineralized film, and **(C)** HDTMS/calcite coating by using methylene blue and soil.

Figure 9 shows the self-cleaning properties of the steel, biomineralized film, and HDTMS/calcite coating, where methylene blue and soil were used as pollutants. After washing with methylene blue solution, the soil particles on the bare steel surface were washed away. However, the methylene blue solution remained on the surface, indicating that the surface exhibited a slight self-cleaning performance for insoluble pollutants but poor self-cleaning performance for soluble pollutants. For the biomineralized film, soil, and methylene blue solution residue were clearly observed on the surface, proving that the biomineralized film exhibited no self-cleaning characteristics for either soluble or insoluble pollutants without superhydrophobic treatment. In contrast, there was no methylene blue residue visible after the HDTMS/calcite coating was washed with the methylene blue solution. This indicates that the HDTMS/calcite coating was superhydrophobic and exhibited good self-cleaning performance. The self-cleaning behaviors of the three surfaces were attributed to the following factors. The bare steel surface was smooth and had some self-cleaning properties for insoluble pollutants, but the hydrophilic surface exhibited no self-cleaning properties for methylene blue. For the hydrophilic biomineralized film, its rough surface allowed the insoluble pollutants to easily attach to and remain on the surface. Moreover, the soluble methylene blue solution readily wet the biomineralized film, causing it to remain on the surface. Owing to the superhydrophobicity of the HDTMS/calcite coating, no soil particles or methylene blue residue remained on the surface. Once the superhydrophobic surface was contaminated, it will soon lose its superhydrophobic properties. Therefore, the good self-cleaning ability ensured the durability of the superhydrophobic coating.

**FIGURE 10:**
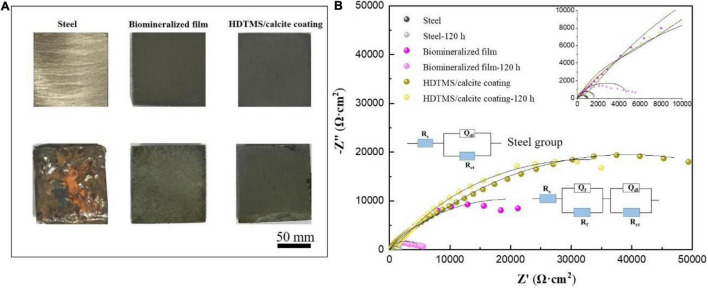
**(A)** Corrosion morphology images and **(B)** electrochemical impedance spectroscopy (EIS) plots of the steel, biomineralized film, and HDTMS/calcite coating before and after 120 h of acetic acid salt spray testing.

The corrosion and mechanical stabilities are two crucial parameters for the practical applications of the coating. We first evaluated the corrosion stability of the HDTMS/calcite coating *via* acetic acid salt spray testing, which was a rigorous corrosion-acceleration test method. After 120 h of acetic acid salt spray testing, the uncoated steel was corroded so severely that the original surface was barely visible. Due to the biomineralized film, the steel was protected significantly more than the bare steel. However, numerous pits were observed on the surface after 120 h of acetic acid salt spray testing. Uniform and pitting corrosion were reduced by the HDTMS/calcite super-hydrophobic coating (Figure 10A). However, the edges of the coating were still slightly damaged, owning to the uneven spraying. In addition, an EIS test was used to investigate the anti-corrosion behaviors of the samples after 120 h of acetic acid salt spray testing (Figure 10B). The fitted data are presented in Table 1. For the steel sample, the diameters of the Nyquist curves were small and decreased regularly with the immersion time. In the presence of a biomineralized film, the Nyquist curve diameters decreased significantly after 120 h of acetic acid salt spray testing. For the HDTMS/calcite coating, the Nyquist curve diameter only decreased slightly, exhibiting high-corrosion stability. To quantitatively compare the differences in the electrochemical parameters of the different groups, the Nyquist plots were fitted using equivalent circuit models, i.e., *R*_s_ (*R*_ct_*Q*_dl_) and *R*_s_ (*R*_f_*Q*_f_) (*R*_ct_*Q*_dl_). The bare steel was best fitted by the *R*_s_ (*R*_ct_*Q*_dl_), whereas the other groups were best fitted by the *R*_s_ (*R*_f_*Q*_f_) (*R*_ct_*Q*_dl_). *R*_s_ represents the solution resistance, *R*_ct_ and *Q*_dl_ represent the charge-transfer resistance and the corresponding double-layer capacitance, respectively, and *R*_f_ and *Q*_f_ represent the film resistance and the corresponding double-layer capacitance, respectively. Constant-phase elements (CPEs) were used instead of capacitors for non-ideal capacitive behaviors, which are defined as follows:


(1) $${\text{Z}}_{\text{CPE}}\left(\omega \right)={\left[Y_{0}{\left(j\omega \right)}^{\text{n}}\right]}^{-1}\left(j^{2}=-1\right),$$

where *Y*_0_ is the CPE amplitude, ω is the angular frequency, and n is the CPE exponent, which is independent of ω. When *n* = 1, *Q* is the pure capacitance. For a capacitance element, the deviation of *n* from unity is due to heterogeneity effects. The common methods for the conversion of CPE parameters to C are those proposed by Mansfeld and Hsu (2001); Sinapi et al. (2008). The barrier protection efficiencies of different strains were also determined using the EIS fitting results of *R*_p_ (*R*_f_ + *R*_ct_) as a function of time. The *R*_p_ values were indicative of the anti-corrosion efficiency. Before acetic acid salt spray testing, the *R*_p_ values for the biomineralized film and HDTMS/calcite coating were 39 and 76 kΩ cm^2^, respectively, which were much higher than those of the bare steel sample (0.4 kΩ cm^2^). After 120 h of acetic acid salt spray testing, the *R*_p_ value of HDTMS/calcite coating decreased slightly to 64 kΩ cm^2^, which was much greater than that of biomineralized film (5 kΩ cm^2^), suggesting a strong protection effect by the super-hydrophobic coating.

**TABLE 1:** Electrochemical impedance parameters from fitted data in Figure 10.

Samples	*R*_s_ (Ω cm^2^)	Y_01_ (S. sec^n^/cm^2^)	*n*	*R*_ct_ (Ω cm^2^)	Y_02_ (S. sec^n^/cm^2^)	*n*	*R*_f_ (kΩ cm^2^)
Steel	3.5	0.7 × 10^–3^	0.8	0.4			
Steel-120 h	7.8	0.5 × 10^–3^	0.9	15	0.9 × 10^–3^	0.9	1.7
Biomineral-film	4.6	8.8 × 10^–6^	0.8	103	0.1 × 10^–3^	0.6	39.4
Biomineral-film-120 h	8.1	0.2 × 10^–3^	0.8	3616	1.2 × 10^–3^	0.5	1.4
HDTMS/calcite	5.5	5.3 × 10^–5^	0.6	2179	3.9 × 10^–5^	0.9	74.5
HDTMS/calcite-120 h	3.3	8.7 × 10^–5^	0.5	1058	9.8 × 10^–5^	0.7	63.5

Even though a biomineralized film can provide perfect micro-/nano-roughness, the reaction between carbonate and acid hinders their applicability as a coating material (Wei et al., 2020). However, HDTMS contains inert and low-surface-energy groups (Huang et al., 2019), endowing treated substrates with extremely low-surface energies and extremely poor wettability (Sachan and Singh, 2019). By using a proper solvent dilution and operation method, HDTMS molecules can penetrate several millimeters into hard and porous inorganic structure materials (Zhang et al., 2018), thus, achieving deep-level long-term hydrophobicity (Geraldi et al., 2018). In the current study, the sprayed HDTMS on the calcium carbonate surface filled in the defects and penetrated the inner calcium carbonate layer. This resolved some of the shortcomings of the biomineralized film for anti-corrosion applications, greatly improving their anti-corrosion potential and application scope (Liu et al., 2018; Guo et al., 2021). Moreover, the improved hardness and roughness of the calcium carbonate layer also improved the mechanical properties and hydrophobicity of the HDTMS layer.

**FIGURE 11:**
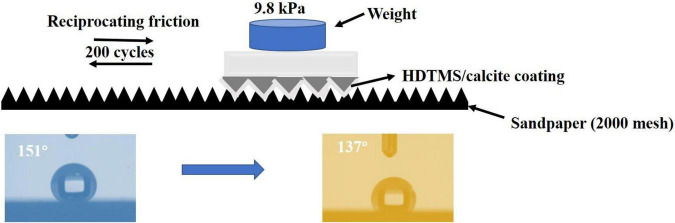
Schematic illustrations of micro-/nano-structure of the HDTMS/calcite coating acting as a solid lubricant during the abrasion by sandpaper (2000 mesh).

The mechanical stability of the HDTMS/calcite coating was investigated by applying reciprocating friction using sandpaper (2000 mesh). After 200 cycles at a 40-cm distance with 9.8 kPa of pressure, the CA of the sample decreased from 151 to 137° (Figure 11). Thus, the HDTMS/calcite coating maintained its hydrophobic properties. One possible explanation for the excellent mechanical stability of the HDTMS/calcite coating was the durability of the biomineralized film, which allowed the micro- and nano-scale roughness to remain when reciprocating friction was applied. In addition, the biomineralized film was composed of many triangular micro- and nano-scale protrusions, which could entrap many hydrophobic organic molecules and reduce the coating damage caused by friction. Although some micro-protrusions were destroyed and then detached during many reciprocating friction processes, the biomineralized film still maintained its overall micro-/nano-structure. The promising mechanical stability and durability of the HDTMS/calcite coating would enable it to work under harsh environments.

## Conclusion

In summary, the biomineralized film was successfully deposited onto steel *via* a biomineralization process using *B. subtilis*. The advantages of this preparation method were environmentally friendly and low cost. The hierarchical micro-/nano-structures of the biomineralized film and the hydrophobicity of the HDTMS endowed the HDTMS/calcite coating with superhydrophobicity, as well as anti-icing and self-cleaning properties. In addition, the HDTMS/calcite coating showed ideal corrosion stability and mechanical durability. Thus, this coating is a promising candidate for practical applications under harsh conditions. In the future, we can further reduce the biomineralization time through a multi-bacterial synergistic effect, thus, promoting the practical application of this method.

## Data Availability Statement

The original contributions presented in this study are included in the article/supplementary material, further inquiries can be directed to the corresponding author.

## Author Contributions

YZ: experiment. NG: Methodology and Software. JK: data curation. ZG: software and validation. JC: visualization and investigation. YY: visualization and investigation. TL: writing – review and editing. All authors contributed to the article and approved the submitted version.

## Conflict of Interest

The authors declare that the research was conducted in the absence of any commercial or financial relationships that could be construed as a potential conflict of interest.

## Publisher’s Note

All claims expressed in this article are solely those of the authors and do not necessarily represent those of their affiliated organizations, or those of the publisher, the editors and the reviewers. Any product that may be evaluated in this article, or claim that may be made by its manufacturer, is not guaranteed or endorsed by the publisher.
